# Influence of Heat Treatment of Nitinol Wire on the Properties of Nitinol/Hybrid Layer for Ibuprofen Release

**DOI:** 10.3390/molecules29215200

**Published:** 2024-11-03

**Authors:** Robert Mroczka, Agnieszka Słodkowska, Jerzy Kubacki

**Affiliations:** 1 Laboratory of X-ray Optics, Department of Chemistry, Institute of Biological Sciences, Faculty of Medicine, The John Paul II Catholic University of Lublin, Konstantynów 1J, 20-708 Lublin, Poland; agnieszka.slodkowska@kul.pl; 2 Faculty of Science and Technology, A. Chełkowski Institute of Physics, University of Silesia in Katowice, 75 Pulku Piechoty 1A, 41-500 Chorzow, Poland; jerzy.kubacki@us.edu.pl

**Keywords:** drug delivery systems, hydroxyapatite electrodeposition, nitinol heat treatment

## Abstract

The efficiency of drug delivery from coatings of metallic implants is one of the key factors. The influence of chemical and thermal treatments of nitinol wire on the corrosion properties, deposition of hydroxyapatite(HA)/poly ε-caprolactone-polyethylene glycol (PEG-*b*-PCL), and the amount of ibuprofen released from that bilayer were studied. The hydroxyapatite layer was electrodeposited by pulse current, while the PEG-*b*-PCL layer was by drop-coating. It was shown that nitinol wire, chemically treated and thermally heated at 470 °C under optimized conditions, is the most optimal substrate for the deposition of uniform and compact hybrid HA/(PEG-*b*-PCL) bilayer. Ibuprofen incorporated into this hybrid bilayer exhibits the maximum release into phosphate-buffered saline (PBS) solution. About 80% of ibuprofen is released within 5 h.

## 1. Introduction

Nitinol wire is a very interesting material that demonstrates unique physicochemical features, such as superelasticity, the shape memory effect, high tensile strength, good corrosion resistance, and biocompatibility [1,2,3,4,5,6], making this material an excellent candidate for the construction of biomedical devices such as dental arch wires, stents, etc. When manufacturing medical devices, the surface of nitinol may undergo different physicochemical changes. From that point of view, it is important to investigate the following aspects: (a) Potential corrosion of the implant in the patient’s body may lead to the release of nickel ions that may be potentially cancerogenic; (b) Under unfavored conditions, corrosion may lead to pitting of surface, reducing the volume of implant and diminishing mechanical properties of the implant. The natural protective layer, consisting of titanium dioxide, forms during nitinol exposure in the water or air. On the other hand, the oxide layer may be non-continuous and can promote pitting. That fact illustrates a strong necessity for optimal chemical and thermal treatments of nitinol to prepare the surface with the greatest anti-corrosion properties. Due to this reason, varied methods of nitinol surface modifications were developed and optimized, such as electropolishing [7,8,9,10,11,12,13], anodizing [11,14,15,16,17], thermal treatment [10,11,12,13,17,18,19,20,21,22], chemical treatment [7,23,24,25,26], and laser surface postprocessing [27,28,29,30].

As a result, a protective titanium dioxide layer is formed. Obtaining better biocompatibility can be achieved by the deposition of the hydroxyapatite layer with a composition of Ca and P close to natural bones or bioglass [14,31,32]. Promising results were received by electrodeposition of HA [23,31]. The HA layer deposited by this method reveals good adhesion and a Ca/P ratio of about 1.67, which is very close to the composition of human bones [23,24,25]. Moreover, in the next step, the HA layer allows the deposit of a subsequent hybrid layer consisting of polymers that can accumulate and release drugs for biomedical applications [25,33]. Different nitinol modification methods on the electrodeposition of the hydroxyapatite layer were studied by Etminanfar et al. [23]. The influence of chemical and thermal treatments at 470 °C under vacuum conditions on the physicochemical properties of nitinol surface was examined. Moreover, the electrodeposition of the hydroxyapatite layer under galvanostatic mode was studied. In further studies [25], electrochemical deposition of the hydroxyapatite layer by pulse current and drug delivery efficiency from the hydroxyapatite/PEG-*b*-PCL bilayer were evaluated. PEG has been successfully used in therapeutic strategies for a long time. PEGylation of proteins or liposomes prolongs blood circulation time, changes biodistribution, and can reduce toxicity and immunogenicity [34,35]. PCL is used in tissue engineering [36,37]. The combination of the hydrophilic PEG and hydrophobic PCL allows a copolymer with higher biodegradability and biocompatibility properties to be obtained [38,39]. The PEG-*b*-PCL copolymers can be used as carriers of various drugs, especially antitumor [40,41,42,43,44] and anti-inflammatory drugs [25,45,46].

However, there are no studies devoted to the influence of thermal treatment at different temperatures under air conditions on the physicochemical parameters and corrosion properties of nitinol wires in combination with the subsequent deposition of hydroxyapatite layer and efficiency of releasing the drug from the hybrid NiTi/hydroxyapatite/PEG-*b*-PCL layer.

In this work, we examined the influence of heating of nitinol wire at 470, 590, and 610 °C under air conditions on morphology, chemistry of surface, and corrosion properties of NiTi. Subsequently, we studied the morphology of the hydroxyapatite layer electrodeposited by pulse current. Consequently, the morphology of bilayer PEG-*b*-PCL/ibuprofen deposited on hydroxyapatite by drop-coating was examined. Finally, the efficiency of ibuprofen released from PEG-*b*-PCL/ibuprofen was evaluated in the context of biomedical application. In the previous studies [23], nitinol wire was annealed at 470 °C under vacuum conditions. Due to this reason, we have chosen that temperature as the basis. We also evaluated nitinol samples annealed at 510 °C and 550 °C and examined them directly using the Raman method. No significantly stronger rutile peaks were observed in comparison to the sample treated at 470 °C. On the other hand, at higher temperatures of 590 °C and 610 °C, due to the formation of significantly thicker TiO_2_, significant rutile peaks were observed), and we decided to select these annealing temperatures (590 °C and 610°C) for further studies. It is worth emphasizing that the deposition of HA and subsequent PEG-*b*-PCL/ibuprofen bilayer on NiTi thermally treated at 590 and 610 °C has not been studied before.

## 2. Results and Discussion

### 2.1. The Effect of Annealing of NiTi

#### 2.1.1. Morphology—SEM and AFM Studies

**Figure 1:**
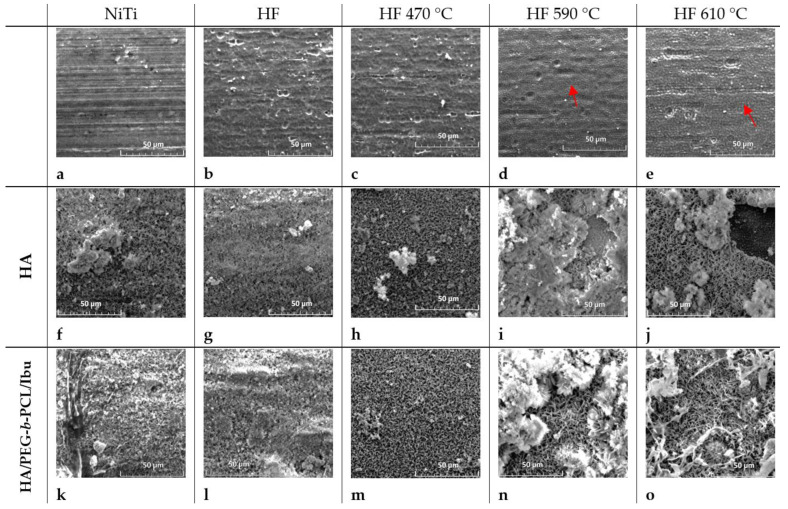
SEM microimages (100 × 100 µm^2^) for (**a**) untreated NiTi; (**b**) etched in HF/HNO_3_/H_2_O; (**c**) heated at 470 °C; (**d**) heated at 590 °C; (**e**) heated at 610 °C; (**f**) hydroxyapatite layer (HA) on NiTi; (**g**) HA on etched nitinol; (**h**) HA on NiTi heated at 470 °C; (**i**) HA on NiTi heated at 590 °C; (**j**) HA on NiTi heated at 610 °C; (**k**) HA/PEG-b-PCL/ibuprofen on untreated NiTi; (**l**) HA/PEG-*b*-PCL/ibuprofen on etched NiTi; (**m**) HA/PEG-*b*-PCL/ibuprofen on NiTi annealed at 470 °C; (**n**) HA/PEG-*b*-PCL/ibuprofen on NiTi annealed at 590 °C; (**o**) HA/PEG-*b*-PCL/ibuprofen on NiTi annealed at 610 °C. Red arrows indicate the porous structure of TiO_2._.

**Figure 2:**
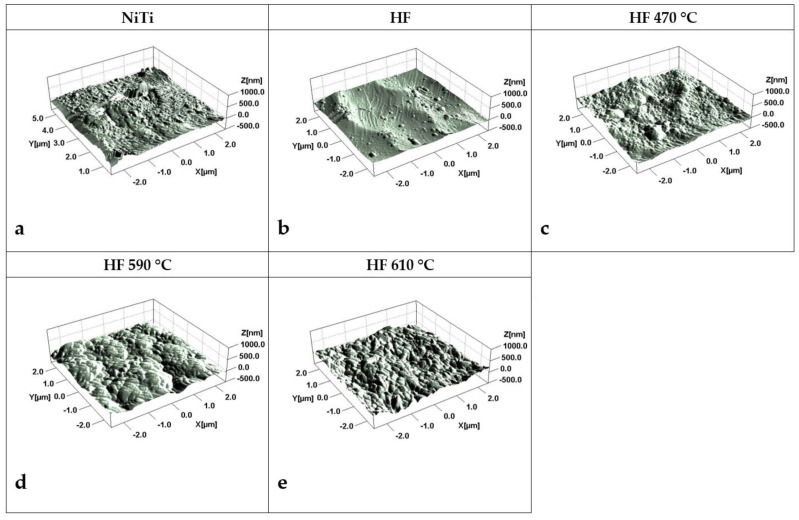
AFM microimages (5 × 5 µm^2^) for (**a)** untreated NiTi; (**b**) etched in HF/HNO_3_/H_2_O solution (abbrev. HF); (**c**) etched in HF/HNO_3_/H_2_O solution and heated at 470 °C for 30 min (HF 470); (**d**) etched and heated at 590 °C (HF 590); (**e**) etched and heated at 610 °C (HF 610).

The effect of chemical treatment and thermal oxidation of NiTi is shown in Figure 1. Inspected samples were assigned as NiTi—(sample untreated), HF—etched in HF/HNO_3_/H_2_O solution, HF_470—etched in HF/ HNO_3_/H_2_O and heated at 470 °C for 30 min, HF_590—etched and heated at 590 °C, and HF_610—etched and heated at 610 °C, respectively. Untreated NiTi wire contains longitudinal and island scratches (Figure 1a). After chemical treatment, the irregular, wavy surface becomes dominant (Figure 1b), which gradually disappears and transforms into a porous structure after thermal treatment (Figure 1c–e) due to the formation of titanium dioxide (indicated by red arrows in Figure 1d,e). Surface roughness obtained from AFM measurements is reduced from Sq = 47.7 nm for untreated NiTi (Figure 2a) to 35.3 nm for the chemically etched surface (Figure 2b). Closer inspection reveals the disappearance of small protrusions on the surface visible for untreated NiTi after etching (Figure 2a and Figure 2b, respectively). Thermal treatment at 470 °C leads to uniform growth of the TiO_2_ layer (Figure 2c) with roughness Sq = 54 nm, while a substantial, wavy feature of etched NiTi substrate is still visible. Annealing at higher temperatures forms a thicker, porous TiO_2_ layer that reveals roughness Sq = 58 nm and Sq = 81 nm for samples treated at 590 °C and 610 °C, respectively. A similar tendency is observed for greater inspection area (20 × 20 µm, Figure S1, Supplementary Materials). Corresponded AFM 2D microimages with height scale are shown in Figure S2 (Supplementary Materials) for comparison.

#### 2.1.2. Chemistry of Surface—XPS Measurements

**Figure 3:**
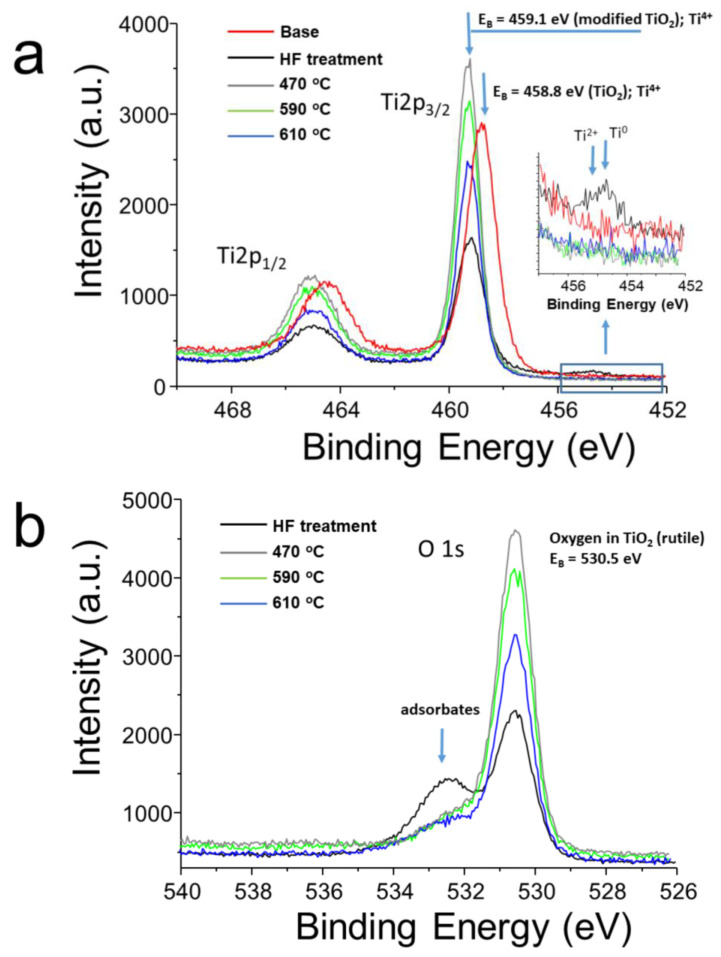
The collection of (**a**) Ti2p and (**b**) O1s photoemission lines recorded for all samples.

In Figure 3a, the collection of the measured Ti2p core lines for all samples has been shown. The peak position of the Ti2p_3/2_ core line for the base sample equals 458.8 eV and is related to the presence of atoms in the TiO_2_ layer, while the modification of the surface by chemical treatment and following elevated heating causes shifts of the other spectra toward higher binding energy of about 0.3 eV. It can indicate the inhomogeneous distribution of titanium oxide on the surface and incomplete oxidation of the tested wires, as indicated by the presence of Ti^2+^ electronic states or Ti^0^ titanium metal. The shape of the O1s spectra has been presented in Figure 3b. All peaks are located at a binding energy of 530.5 eV and indicate the modified surface of the suggested TiO_2_ layer after chemical and temperature treatments of study wires in relation to clean titanium dioxide. An additional bump marked by arrows indicates additional components presented in the shape of the O1s line, which can be related to adsorbates. The influence of chemical treatment and the annealing process at different temperatures clearly changes the intensity of the core levels. The sequence of intensity changes is identical in both cases of studied lines Ti2p and O1s. The lowest intensities of Ti2p_3/2_ and O1s peaks are observed in the base and the acid-etched samples. The annealing process at 470 °C leads to an increase in their intensities, while subsequent annealing steps at 590 °C and 610 °C led to a gradual decrease. Additionally, for the Ti2p_3/2_ line, successive annealing steps remove Ti^2+^ and Ti^0^ electronic states from the surface of the tested materials, as shown in the enlarged inset in Figure 3a. The annealing process does not significantly influence the shape of the Ti2p doublet. In the case of the O1s line, an additional feature is observed at higher binding energy for the chemically etched samples at room temperature conditions (RT conditions), which can be attributed to the presence of adsorbates on the surface. The intensity of these features significantly decreases during the annealing process at particular temperatures.

**Figure 4:**
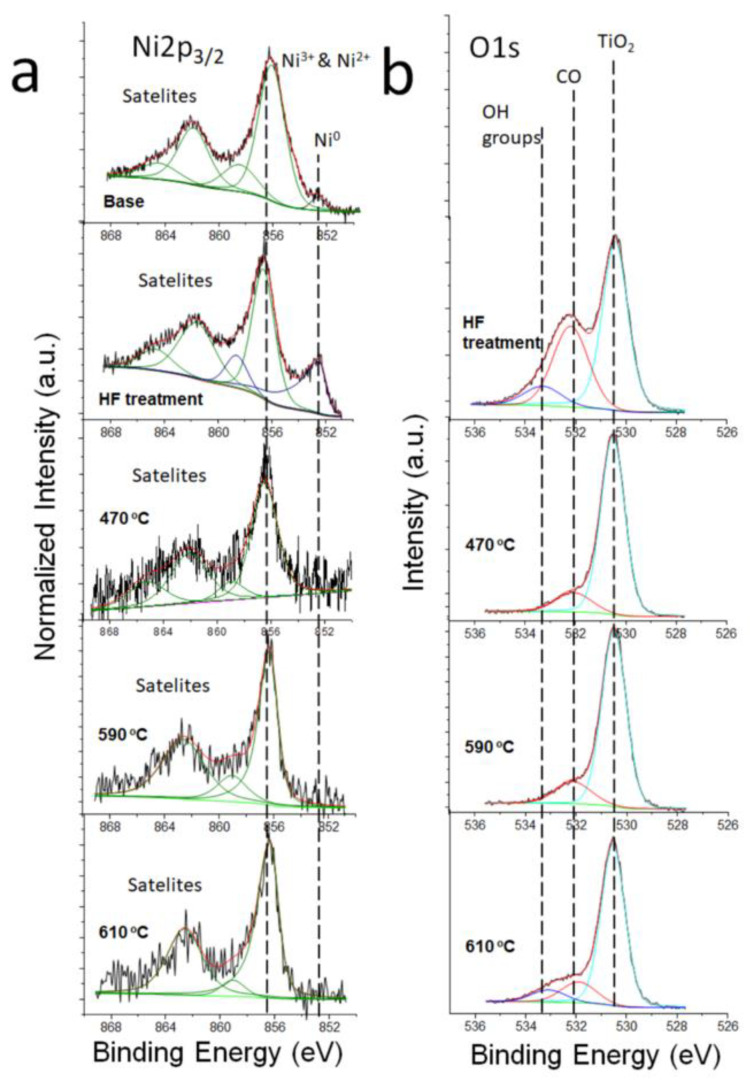
Deconvoluted spectra of Ni2p_3/2_ (**a**) and O1s (**b**) core lines at different preparation procedures.

Figure 4a,b show the deconvolution of the Ni2p_3/2_ and the O1s core lines for all measured samples. The shape of Ni2p_3/2_ photoemission lines contains several components located at 852.6 eV and 856.2 eV and others at higher binding energies. The binding energy of the first component indicates the presence of a metallic state of nickel or NiTi bond. The position of the second component indicates the presence of Ni^2+^ ions, as was shown by Gu et al. [47]. However, Ni2p spectra are complex and contain many peaks, and assigning specific binding energies to particular electronic states is very difficult. For example, the positions of the main peaks of the Ni2p_3/2_ state for NiO (Ni^2+^), Ni(OH)_2_ (Ni^2+^), and NiOOH (Ni^3+^) related to ions located at 854.7, 855.3, and 855.8 eV, respectively, were investigated [48]. In turn, Krajewski et al. [49] reported the presence of Ni^3+^ (Ni_2_O_3_) and Ni^2+^ Ni(OH)_2_ ionic states at 856.6 eV and 855.3 eV for NiFe nanochains. In our opinion, the presence of nickel hydroxide on chemically- and temperature-modified ex situ surfaces cannot be excluded. Hence, we suggest relating the position of the 2p_3/2_ line at 856.2 eV to the presence of Ni^2+^ and Ni^3+^ ions on the studied surfaces.

The metallic states are present in the base and chemically etched samples, while mixed Ni^2+^ and Ni^3+^ states exist in all samples. The O1s line contains three components attributed to the presence of oxygen atoms in the TiO_2_ compound and adsorbed CO molecules and OH groups. The component attributed to OH groups disappears during the annealing processes at 470 °C and 590 °C and reappears during the final annealing step at 610 °C. The compositions of Ti, Ni, and O are listed in Table 1.

**Figure 5:**
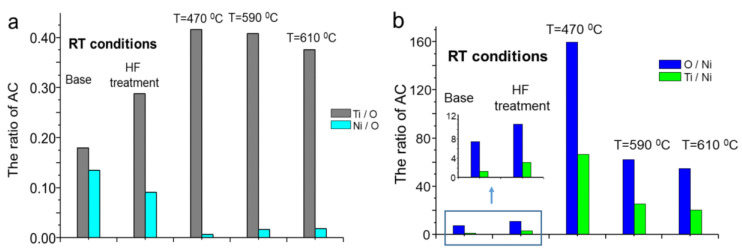
The atomic concentration ratios calculated from the core levels recorded obtained for Ti/O and Ni/O (**a**) and O/Ni and Ti/Ni (**b**) change after different preparation procedures. Abbreviation RT means room temperature.

In Figure 5a, the ratios of calculated atomic concentration of Ti/O and Ni/O for each annealing step are shown. Initial chemical treatment contributes to a visible increase in the Ti/O ratio and a decrease in Ni/O. The first step of annealing under normal conditions for T = 470 °C results in a drastic increase in the Ti/O ratio and a decrease in Ni/O. The next temperatures of annealing at 590 °C and 610 °C resulted in a gentle decrease in Ti/O and a simultaneous increase in Ni/O. An annealing at 610 °C leads to a more significant decrease in Ti/O. A slightly greater Ti/O ratio after thermal heating was also observed by Chrzanowski et al. [50], where the Ti/O ratio increases from 0.31 to 0.35 (slightly lower than in our case). While that fact was not commented on, we assume that a slightly greater ratio of Ti/O and Ni/O after thermal heating at 470 °C corresponds to the composition of the rutile phase that is not observed for non-heated etched NiTi.

We suppose that the ability to measure changes in electron structure with the operando (real-time) X-ray absorption technique during annealing will make it possible to determine the contribution of electrons to the redox potential. We are planning this kind of measurement in the future at the SOLARIS National Synchrotron Radiation Centre in Cracow.

For all applied temperatures, decreasing Ti/Ni and O/Ni ratios can be observed, as shown in Figure 5b.

At the first temperature of 470 °C, the changes in Ti/O and Ni/O ratios can be described as the escape of adsorbates from the environment of Ti and Ni atoms and the formation of active areas. In the following temperatures of 590 °C and 610 °C, we can suppose the increase in adsorption of oxygen from the atmosphere and oxidation of the surface. An increase in the amount of oxygen at the surface leads to a decrease in the Ti/O ratio. The ratio Ti/Ni is a very important factor that allows the estimation of how the surface can be safe in terms of the release of nickel ions. The Ti/Ni ratio is the lowest (1.34) for untreated NiTi wire. After chemical etching, the Ti/Ni ratio increases 2.35 times. The surface of chemistry after etching is determined by the three main reactions [1].

Dissolution of dioxide TiO_2_ layer by hydrofluoric acid

(1) $${TiO}_{2}+6F^{-}+{4H}^{+}\to {\left\lfloor {TiF}_{6}\right\rfloor }^{2-}+{2H}_{2}O$$

In the second reaction, oxidation of titanium occurs

(2) $$Ti+{4NO}_{3}^{-}+{4H}^{+}\to {TiO}_{2}+{4NO}_{2}+{2H}_{2}O$$

Nickel present in nitinol alloy is dissolved in the acid-releasing nickel ions:

(3) $$Ni+{2H}^{+}\to {Ni}^{2+}+H_{2}$$

The latter reaction is more energetically preferred over reactions (1) and (2) and stands for lower nickel concentration after chemical etching [1,11,51,52].

#### 2.1.3. Chemistry of Surface—TOF-SIMS Measurements

**Figure 6:**
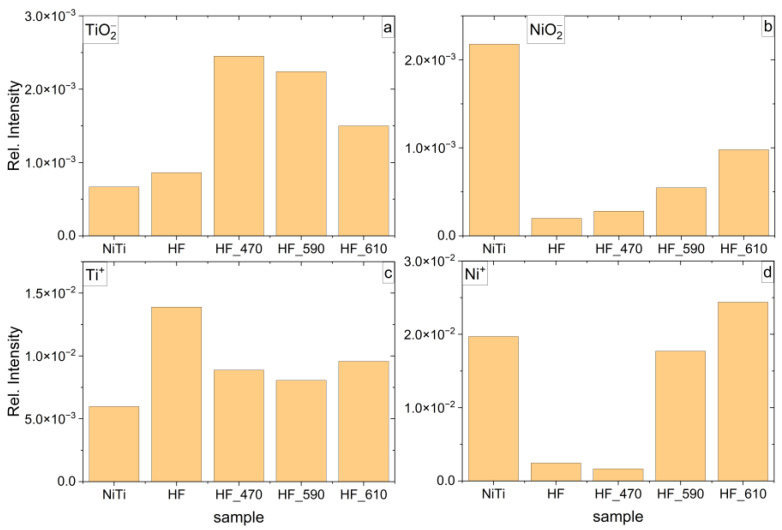
Distribution of relative intensity of negative and positive ions (**a**) TiO_2_^−^, (**b**) NiO_2_^−^, (**c**) Ti^+^, and (**d**) Ni^+^ identified in TOF-SIMS spectra for untreated NiTi (assigned in Figure 6 as NiTi), etched in HF/HNO_3_/H_2_0 (assigned as HF), etched and annealed at 470 °C (assigned as HF_470), etched and annealed at 590 °C (assigned as HF_590), and etched and annealed at 590 °C (assigned as HF_590).

The TOF-SIMS technique is a surface-sensitive method that characterizes 2–3 nm of the top surface layer. Moreover, this method can be applied to estimate titanium dioxide layer thickness on NiTi by employing a sputtering source [10,53,54]. On the other hand, XPS characterizes the chemistry of 3–10 nm of the top surface depending on the density of the material. Similarly to TOF-SIMS, the XPS method can be used for TiO_2_ thickness analysis [1,47,50,55,56]. The intensity distribution of selected positive and negative ions of five evaluated samples assigned as NiTi, HF, HF_470, HF_590, and HF_610 is shown in Figure 6. The intensity of Ti^+^ (Figure 6c, we assume that it is mainly titanium in a metal state) demonstrates the greatest value for HF-treated NiTi, which is determined by reactions (1) and (2). The occurrence of Ti^+^ that may correspond to metallic Ti for NiTi, HF_470, HF_590, and HF_610 is not supported by XPS measurements. As the SIMS method does not allow identification of the oxidation state of elements, it seems that the Ti^+^ ion is yielded from TiO_2_, as well as from Ti in the metal state (sample HF). Higher intensity of Ti^+^ for the HF sample may suggest a dominant ratio of the metallic form of Ti for that sample, while accurate quantification and direct oxidation state can be estimated only from XPS data. TiO_2_^−^ ion (Figure 6a) may be assigned to the TiO_2_ dioxide layer as we observe significantly greater intensity for HF_470, HF_590, and HF_610 samples. The distribution of the TiO_2_^−^ intensity is roughly similar to the Ti concentration estimated by XPS (Table 1) while demonstrating greater deviation.

**Figure 7:**
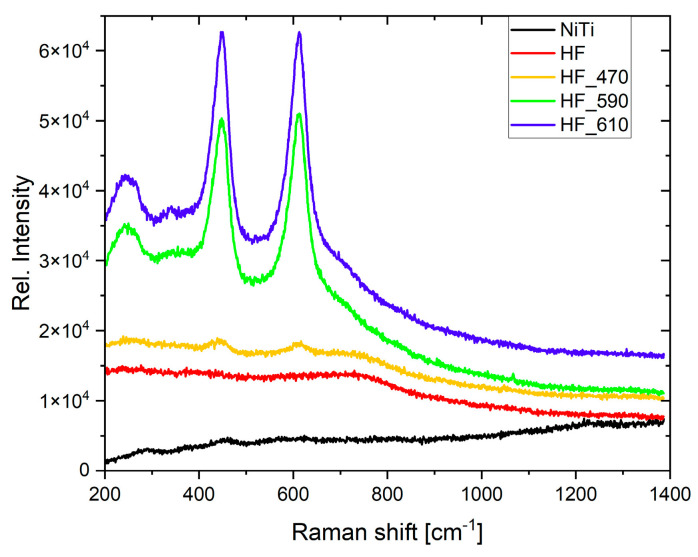
Raman spectra for (untreated NiTi; etched in HF/HNO_3_/H_2_O solution (abbrev. HF); etched in HF/HNO_3_/H_2_O solution and heated at 470 °C for 30 min (HF_470); etched and heated at 590 °C (HF_590); etched and heated at 610 °C (HF_610).

The intensity distribution of Ni^+^ (Figure 6b) and NiO_2_^−^ (Figure 6d) shows the greatest value for NiTi (untreated). After etching, the lowest intensity of NiO_2_^−^ is observed for the HF sample, while Ni^+^ is for HF_470. Similarly, as it is for Ti, it is not possible to distinguish the chemical state of Ni by TOF-SIMS. The similar intensity of Ni^+^ and NiO_2_^−^ for HF and HF_470 samples is contrary to the XPS data that show significantly higher Ni concentration for the HF sample (Table 1). It is determined by the fact that the nickel oxide layer for the untreated NiTi sample is thicker than for the HF sample. In consequence, the XPS cumulative content of Ni is considerably greater for NiTi (untreated) than for the HF sample (Table 1). This observation may suggest that the thickness of the Ni/NiO_2_/Ni_2_O_3_ layer for the HF sample is in the order of 2–3 nm. After heat treatment, the intensity of NiO_2_^−^ increases roughly linearly with temperature (samples HF_470, HF_590, HF_610), and it is roughly consistent with XPS data (Table 1), while the intensity of Ni^+^ demonstrates very high enhancement for HF_590 and HF_610. The latter abnormal observation strongly suggests that this effect may be determined by the fact that nickel oxide resides in thick, porous TiO_2_^−^ dioxide, the rutile phase (see Raman data, Figure 7) that strongly increases the yield of Ni^+^ ion.

#### 2.1.4. Raman Studies

The formation of TiO_2_ is evidenced on Raman spectra (Figure 7). The most dominant bands at 232 cm^−1^, 446 cm^−1^, and 611 cm^−1^ correspond to the rutile phase of TiO_2_ [47,53]. The intensity of peaks is roughly proportional to the thickness, roughness, and porosity of the TiO_2_ layer [57]. For HF_590, which reveals thinner layer thickness, enhancement of the Raman signal can be determined by smaller grains (notice Figure 2, AFM results) [58]. In consequence, the Raman signal is comparable to sample HF_610, which exhibits thicker layer thickness. For untreated and chemically etched nitinol wires, no visible peaks are observed. Moreover, atanase peaks (bands 515.5 cm^−1^ and 636.7 cm^−1^) are not observed, as it was reported for multicycle short–fast heating and cooling NiTi [47]. The nickel oxide phase was not observed. The latter fact does not exclude the presence of a nickel oxide layer that is very thin and resides inside thick TiO_2_. Due to this reason, it cannot be detected by Raman spectroscopy [47], but it can be revealed by XPS.

#### 2.1.5. Elemental Analysis—SEM-EDS Studies

**Figure 8:**
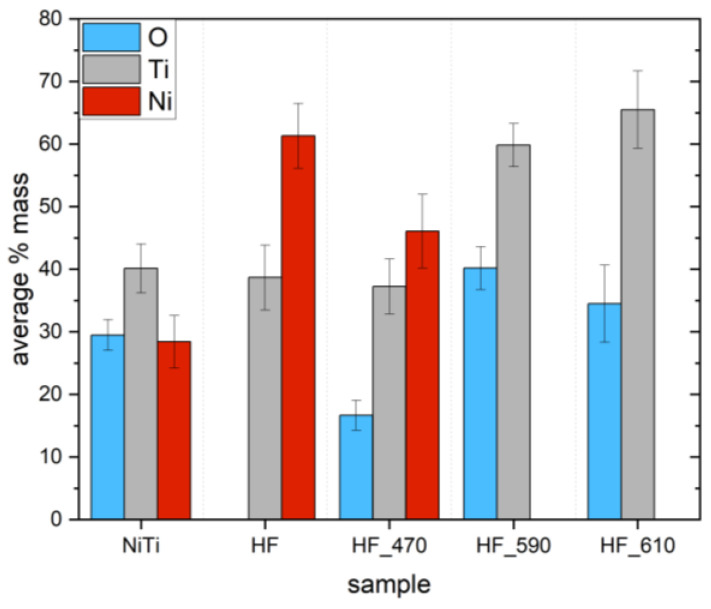
Distribution of elements for (untreated NiTi; etched in HF/HNO_3_/H_2_O solution (abbrev. HF); etched in HF/HNO_3_/H_2_O solution and heated at 470 °C for 30 min (HF_470); etched and heated at 590 °C (HF_590); etched and heated at 610 °C (HF_610).

Element analysis was determined by the SEM-EDS method. Measurements were carried out in six points on the sample. The mass content for O, Ni, and Ti for untreated nitinol (NiTi) is shown in Figure 8. The presence of oxygen is determined by the light oxidation of nitinol by the manufacturer. The appearance of the native layer of nitinol (untreated) was dark. After chemical etching, the oxygen disappears from the surface, and the concentrations of Ni and Ti are 55 and 45% (mass), while the surface becomes light. Thermal heating leads to a gradual increase in oxygen content and a decrease in the ratio of Ti/Ni due to the increasing thickness of the TiO_2_ layer due to oxidation. For samples HF_590 and HF_610, Ni completely disappears. The basic difference between the composition obtained by XPS and EDS is determined by different penetration depths. XPS is a highly surface-sensitive technique limited to a maximum of 10 nm at the top of the surface in a vertical direction, while EDS is a rather bulky technique with a penetration depth of up to 1–2 µm.

Due to this reason, XPS data represent the composition of the thin layer and nearly perfectly corresponds to the composition of the outermost top thin TiO_2_ layer, while EDS composition (Figure 8) is strongly determined by the underlayer of NiTi bulk substrate. It explains the fact that Ni completely disappears for samples annealed at 590 and 610 °C.

The thickness of TiO_2_ was not determined in our studies, while other investigations showed that after 1h annealing of polished NiTi, TiO_2_ layer thickness was equaled to 1050 nm [59]. For example, after etching (sample HF), the composition determined by EDS (Figure 8) is well suited to the manufacturer specification (Ni 55%, Ti 45%), while XPS data show a significant diminishing of Ni compounds in the outermost top layer due to partial oxidation of Ti to TiO_2_ under ambient conditions (room temperature). In this case, TiO_2_ thickness is as low as 0.5 nm [59].

#### 2.1.6. Corrosion Behavior of NiTi Wires

**Figure 9:**
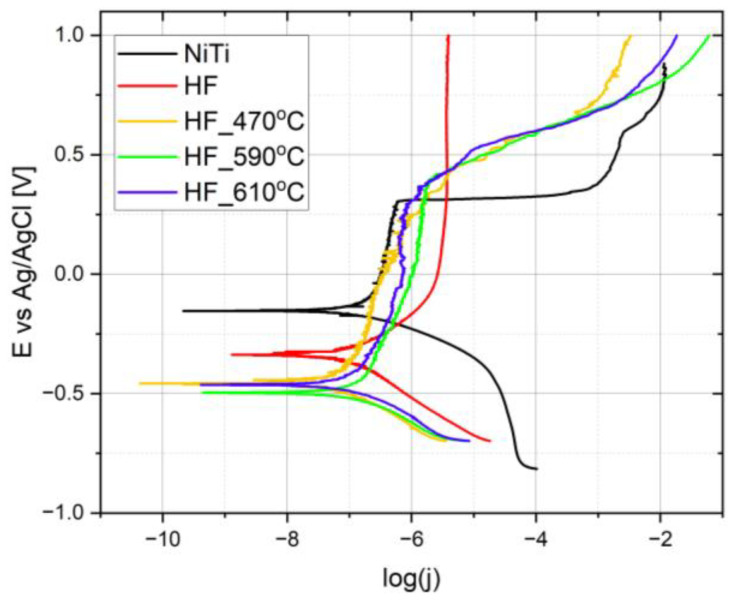
Polarization curves for (untreated NiTi; etched in HF/HNO_3_/H_2_O solution (abbrev. HF); etched in HF/HNO_3_/H_2_O solution and heated at 470 °C for 30 min (HF_470); etched and heated at 590 °C (HF_590); etched and heated at 610 °C (HF_610).

Figure 9 shows polarization curves carried out in PBS solution. Corrosion parameters calculated from the linear Tafel slope are listed in Table 2.

The smallest corrosion current (I_c_ = 18.8 nA/cm^2^) among the evaluated samples was calculated for NiTi heated at 470 °C, while the highest corrosion current was calculated for untreated nitinol (I_c_ = 44.1 nA/cm^2^).

Among the thermally heated samples, it is clearly observed that the higher the temperature, the greater the corrosion current, while the corrosion rate is similar for HF_470 and HF_590.

Distinct breaking potential for the passive region is observed for untreated and thermally heated samples and not for the chemically treated NiTi sample. Untreated nitinol demonstrates the shortest snap of the passive region (ranges from −0.15 to +0.3 V), with a breaking potential around +0.3 V. For greater potentials, the passive layer is rapidly destroyed, and current increases very fast up to 10^−3^ A/cm^2^. For potentials greater than +0.4 V, a transpassive layer is gradually formed. After etching, no breaking potential is observed for the evaluated potential range. The passive layer is clearly observed for potentials greater than +0.15 V, while the current in this region reaches the greatest value. It is the consequence that for potentials located below the passive barrier (E < −0.3 V), the surface is absent of TiO_2_ due to etching that is finally formed in the passive region. Thermally treated samples exhibit passive regions up to around +0.3 V, similar to untreated nitinol, while for greater potentials, transpassive regions with similar current density are clearly seen. For the greatest potentials (E > +0.75 V), a higher current is observed for HF_590 and HF_610 than for HF_470. Similar behavior occurs in the passive region (−0.3 V < E < + 0.3V). For sample HF_470, the TiO_2_ layer is thinner than for HF_590 and HF_610, while it is more compact and less porous, which provides a slightly better anti-corrosion barrier. We assume that the combination of thickness, porosity (Figure 2c), and composition of the TiO_2_ layer (determined by XPS and TOF-SIMS) formed on etched NiTi and heated at 470 °C is optimal for forming a compact and homogenous hydroxyapatite layer. Less porosity (lower roughness, Figure 2c) allows the maintenance of less porosity of the deposited HA layer. In consequence, a smoother and more uniform HA layer maintains more uniform penetration of PEG-*b*-PCL into HA due to better wettability, which is determined by lower roughness. It suggests that the morphology of the electrodeposited HA layer is strongly determined by the chemical composition and morphology of the substrate, like other electrodeposition processes, such as copper electrodeposition on nitinol wire [60]. It determines lower corrosion current. On the other hand, thicker TiO_2_ for HF_590 leads to generally the same corrosion properties as for HF_470 (corrosion rate 0.0002 mm/year).

In comparison to the previous studies [56], the corrosion current for the untreated NiTi was one order of magnitude greater (j_c_ = 4.41 × 10^−8^ A/cm^2^), while other investigations reported one order of magnitude greater corrosion current (j_c_ = 34 × 10^−8^ A/cm^2^) [59]. After the anodizing current was reduced to j_c_ = 1.5 × 10^−7^ A/cm^2^ and after annealing at 400 °C for 1 h, it further reduced to j_c_ = 1.9 × 10^−8^ A/cm^2^. The latter results are still slightly greater than for nitinol chemically etched and annealed at 470 °C. In other investigations [61], chemical treatment in FeCl_3_ and subsequent annealing at 400 °C for 1 h led to a corrosion current equal to 8.6 nA/cm^2^, which is comparable to sample HF_470.

Milosev et al. [62] reported corrosion currents of 1∙10^−8^ and 0.5 ∙10^−8^ for previously polished NiTi annealed at 500 and 600 °C, respectively.

The dependence of OCP (open circuit potential) after immersing in PBS solution (before the determination polarization curve) is shown in Figure S3 (Supplementary Materials). Untreated and chemically etched nitinol exhibit similar OCP curves that gradually increase and finally reach a steady state after 570 s (dE/dT < 1 µV/s), while OCP potential for untreated NiTi is positively shifted of 0.2 V. It arises from the fact that untreated nitinol possesses thin TiO_2_ layer created during the manufacturing process. After thermally heating at 470 °C, the potential is shifted toward more negative values, and after about 100 s, it reaches a more stable value, while the assumed steady state limit (dE/dE < 1 µV/s) does not reach up to 1200 s. For samples HF_590 and HF_610, the starting potential is the most positive, which gradually shifted toward more negative values. It may arise from the porosity layer and higher content of nickel in the outermost part of the TiO_2_ layer.

**Figure 10:**
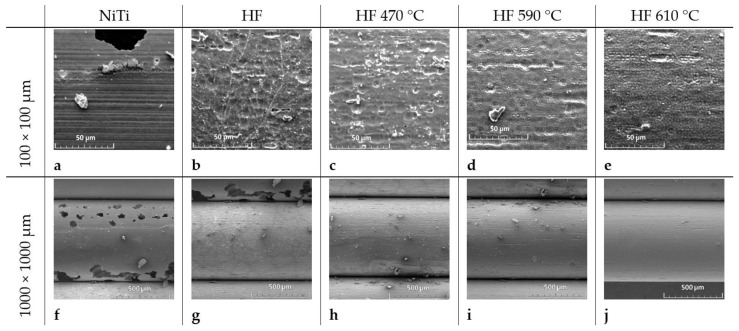
SEM microimages for (**a**,**f**) untreated NiTi; (**b**,**g**) etched in HF/HNO_3_/H_2_O solution (abbrev. HF); (**c**,**h**) etched in HF/HNO_3_/H_2_O solution and heated at 470 °C for 30 min (HF_470); (**d**,**i**) etched and heated at 590 °C (HF_590); (**e**,**j**) etched and heated at 610 °C (HF_610).

After withdrawing wires from the PBS solution, the morphology surface demonstrates significant differences (Figure 10). For untreated NiTi (Figure 10a), pitting corrosion occurs due to localized enhanced dissolution of Ni according to the reaction

(4) $$Ni={Ni}^{2+}+2e^{-}$$

Pitting corrosion is driven by the breaking of transpassive potential, oxygen evolution, and transpassive dissolution of NiTi [7].

The preferential dissolution of Ni was supported by the distribution of Ni^+^ and Ti^+^ on the surface determined by TOF-SIMS. For sample HF, pitting corrosion is not observed (polarization curve does not reveal breaking potential), while due to relatively high corrosion current, small cracks on the surface are visible (Figure 10b). Sample HF_470 (Figure 10c) exhibits very small protrusions similar to HF_590. The highest quality demonstrate surface was treated at 610 °C (Figure 10e,j). In the latter case, the TiO_2_ porous structure is preserved, which means that the dissolution of nickel (reaction 4) is very uniform and does not destroy the TiO_2_ protection layer.

#### 2.1.7. Deposition of Hydroxyapatite Layer (HA)

The hydroxyapatite layer was electrodeposited by pulse current (see Section 3). Electrochemical reactions that occur on the working electrode (cathode) that lead to the formation of hydroxyapatite can be found elsewhere [31]. The deposited hydroxyapatite layer (thickness ~10 µm, which was determined by laser scan micrometer) reveals relatively uniform morphology for etched and annealed at 470 °C, while the higher nonhomogeneous structure of HA becomes more dominant for samples NiTi (untreated), HF_590, and HF_610 (Figure 1). Moreover, uncovered places of NiTi by the HA layer can be noticed for the sample heated at 610 °C. The latter fact can be determined by a thick porous TiO_2_ layer that locally may block electrodeposition HA. The ratio of Ca/P was determined by SEM/EDS and is tabulated in Table 3 (oxygen concentration was omitted). Lower concentrations of P and Ca for sample NiTi (untreated) may be determined by some contamination of the surface by C and Na. The ratio Ca/P is around 1.70, which is very close to the typical composition of the hydroxyapatite layer. A slightly higher Ca/P ratio for HF_610 may suggest that some other phases of Ca/P contained more Ca than P that can be incorporated into HA for those samples. In recent studies [31], HA obtained under galvanostatic conditions reveals a Ca/P ratio of around 1.67 for greater current densities. In our studies, applied pulse current with low current densities similar to the recent studies [25] allows a good quality of the HA layer to be obtained, with a Ca/P ratio like natural HA.

#### 2.1.8. Releasing of Ibuprofen from HA/PEG-b-PCL

A total of 4 mg/cm^2^ of ibuprofen dissolved in PEG-*b*-PCL solution (0.15%) was loaded onto a hybrid HA/PEG-*b*-PCL (12 µg/cm^2^) layer (total area 0.5 cm^2^) by drop-coating. Morphologies of the layer deposited on NiTi(untreated)/HA/PEG-*b*-PCL, HF, HF_470, HF_590, and HF_610 after evaporating of solvent are shown in Figure 1k–o, respectively. The most homogenous and compact structure is observed in a layer of HA/PEG-*b*-PCL/Ibu deposited on HF_470 substrate. On the other hand, significant protrusions are observed for the HA/PEG-*b*-PCL/Ibu layer deposited on HF_590 and HF_610. The morphology of all HA/PEG-*b*-PCL/Ibu layers is roughly very similar to the hydroxyapatite layer. We can hypothesize that the morphology of HA does not change after the deposition of the PEG-*b*-PCL/Ibu layer.

**Figure 11:**
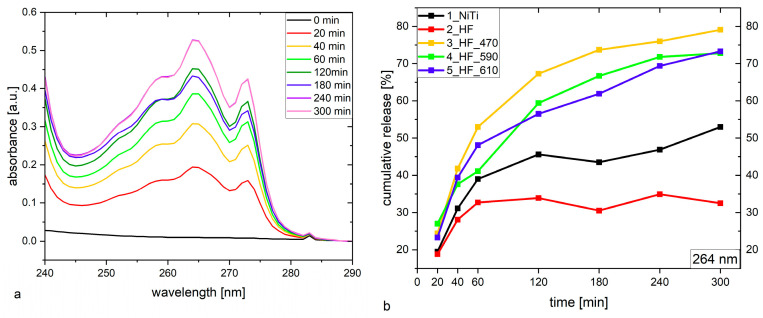
(**a**) UV-VIS curves for ibuprofen released from hybrid layer HA/PEG-*b*-PCL deposited on NiTi(untreated); (**b**) Cumulative release % of ibuprofen from HA/PEG-*b*-PCL deposited on HA/NiTi, HA/HF, HA/HF_470, HA/HF_590, and HA/HF_610.

UV-VIS spectra (Figure 11a, and Figure S4, Supplementary Materials) show that the amount of ibuprofen released into the PBS solution exponentially increases during 300 min of the experiment, reaching 80% (Figure 11b) of the released amount for nitinol wire heated at 470°C. Heating at higher temperatures (590 and 610 °C) decreases the total amount of ibuprofen to ~73%. Cumulative ibuprofen release was calculated based on the reference curve (Figure S5, Supplementary Materials). Delivery of ibuprofen from hybrid layer HA/PEG-*b*-PCL deposited on non-thermally treated nitinol leads to a significantly reduced amount of ibuprofen released. It can be determined by molecular rearrangement and roughness of the HA layer that likely causes stronger binding of ibuprofen molecules inside HA/PEG-*b*-PCL.

The manufacturer of ibuprofen specifies that its solubility in PBS solution is 2 mg/mL [63]. We used 3 mL of buffer for the UV-VIS experiment (the volume of glass cuvette), which allows the dissolving of 2 mg of ibuprofen to maintain the sink condition (1/3 of solubility). We loaded 2 mg of ibuprofen on the NiTi/HA/ PEG-*b*-PCL bilayer, so the silk condition was fulfilled. In our drug release experiments, we conducted tests on the HF/HA/PEG-*b*-PCL sample, incorporating fresh PBS solution at regular intervals. This approach allowed us to generate cumulative release profiles over time, as shown in the figure below, corresponding to the data presented in Figure 11b. However, our objective was to more accurately simulate physiological conditions where body fluids are not replenished discretely, stepwise. Due to this reason, we did not refresh the PBS solution during the UV-VIS experiment. If we compare Figure S6 (Supplementary Materials, NiTi_etched/HA/PEG-*b*-PCL immersed into PBS refreshed) with results obtained for the NiTi_etched/HA/PEG-*b*-PCL immersed into PBS non refreshed) sample denoted as 2_HF in Figure 11b, we can observe that up to 60 min of ibuprofen release is similar, while longer time for the non-refreshed PBS solution, Ibu is not released. For fresh PBS solution, it is released up to 50% at 240 min. This means that the HA/ PEG-*b*-PCL bilayer deposited on etched NiTi can limit the capabilities of Ibu release for non-refreshed PBS solution while it is maintained for refresh PBS. In our opinion, it is better to assume that body fluid is not continuously refreshed in an ideal way. Under these circumstances, the experiment with a non-fresh PBS solution better simulates real conditions.

On the other hand, our results show that ibuprofen delivery into PBS solution is considerably faster than it was previously studied [25], where ibuprofen was loaded on the thicker layer of PEG-*b*-PCL (0.3 mg/cm^2^), and the total time of releasing was 20 h. That may be related to the mass ratio of ibuprofen to PEG-*b*-PCL equal to 100:1 in our mixture, while Etminanfar et al. [25] used a mixture with a ratio of 20:1.

The spectra recorded at different intervals of time are very similar. This means that molecules of ibuprofen are stable in PBS during release.

We assumed that ibuprofen released from the HA/PEG-*b*-PCL bilayer would be used only for reducing pain or potential symptoms of inflammation after the implementation of an implant in the body of a patient. Due to this reason, releasing 70% of ibuprofen within 5 h after implant insertion surgery should be optimal for that purpose. Similar assumptions were realized in the recent studies devoted to the release of ibuprofen from the HA/PEG-*b*-PCL bilayer [25]. In this term, such fast release of ibuprofen coincidence with releasing of ibuprofen from acrylic matrix tablets made up of EE, ((poly-(n-butyl methacrylate-(2-dimethylaminoethyl)-methacrylate-methyl methacrylate) copolymer with ratio 1:2:1) that releases 55% of IBU within 4 h, while EL (ethyl acrylate–methacrylic acid copolymer with ratio 1:1) releases 100% of IBU after 2.5 h. EE polymer is soluble under acidic conditions (pH < 6.0), while EL is soluble in a neutral and base environment (pH > 5.5). Due to this reason, interpolyelectrolyte complex (IPEC) consists of a combination of EE and EL that is capable of releasing 55% of IBU after 4 h and can be a good candidate to stimulate the gastrointestinal tract, with the first hours releasing IBU into acidic environment and the next 2 h into buffer condition (pH = 6.8) [64]. In the context of this application, we can apply the whole IBU to the buffer condition around implant space in the body, which is an additional benefit in comparison to the tablet made up of IPEC. Moreover, the release of paracetamol from polymer matrix aceclofenac (ACE) and diclofenac sodium (DIS) in the form of tablets can also be extended up to 24 h [65].

## 3. Materials and Methods

**Figure 12:**
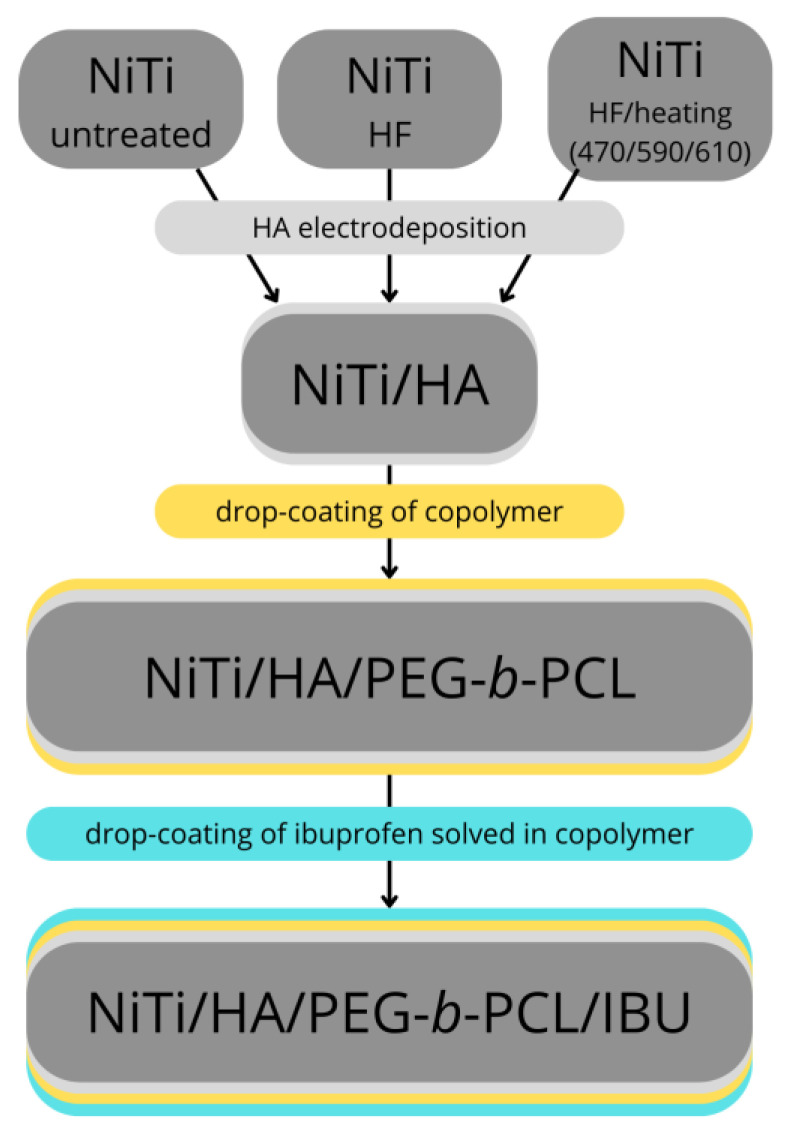
General scheme of experiment.

The general scheme of the experiment is shown in Figure 12.

### 3.1. Nitinol Surface Preparation

NiTi alloy (0.66 mm, 15 cm, cold draw, dark oxidized, Ni 54.7–57%, Ti 43–45.5%, EUROFLEX GmbH, Pforzheim, Germany) was used as a substrate. Chemical etching of the wire was conducted in an acid bath consisting of HNO_3_/HF/H_2_O = 4:1:5 (volumetric ratio) for 4 min. After, etching wires were immersed in boiling deionized water for 10 min. Oxidation of wires was carried out in the furnace in the air atmosphere at 470, 570, and 610 °C for 30 min. Before heat treatment, wires were inserted into borosilicate capillaries (internal diameter 1 mm, outer diameter 5 mm, Simax, Praha, Czech Republic). It is crucial for optimized thermal heating and allows the transfer of heat gradually into the nitinol wire and, in consequence, maintains superelasticity and mechanical properties.

### 3.2. Corrosion Behavior of NiTi Wires

Electrochemical measurements were conducted in phosphate-buffered saline (PBS), pH~7.2, solution at 37 °C. Potentiostat/galvanostat AUTOLAB PGSTAT 128N (EcoChemie, Utrecht, Netherlands) was employed for recording potentiodynamic curves. The electrochemical setup consisted of a glass cylinder (100 mL), examined NiTi wires served as the working electrode, platinum wire as a counter electrode, and miniature Ag/AgCl electrode (leakless ET072, eDAQ, Sydney, Australia) as the reference electrode, respectively. Potentiodynamic curves were recorded from −0.70 to 1.0 V (in relation to OCP). OCP was determined for 1200s (unless changes in the oscillation of OCP potential (dE/dt) reached a value lower than 1 µV/s).

### 3.3. Electrochemical Deposition

All the reagents used were analytical grade, and the water was distilled and deionized. The electrodeposition bath was prepared using 8.4 mM of Ca(NO_3_)_2_·4H_2_O (Avantor Performance Materials, Gliwice, Poland S.A.), 5 mM of NH_4_H_2_PO_4_ (Sigma-Aldrich, Saint Louis, MO, USA), 0.1 M of NaNO_3_ (Chempur, Piekary Śląskie, Poland), and 6 mL/L H_2_O_2_ (Chempur, Poland) [25]. To achieve the desired working pH of 6.0, 0.1 M Tris (Sigma-Aldrich, Saint Louis, MO, USA) solution was added. Potentiostat/galvanostat AUTOLAB PGSTAT 128N (EcoChemie, Utrecht, Netherlands) was employed for the control of the process and recoding chronopotentiometry curves. The electrodeposition of HA was performed using a regular two-electrode setup: the NiTi wire served as the cathode and platinum cylindric mesh as the anode (diameter 5 cm) mounted in a 250 mL glass cylinder. The electroplating bath (200 mL) was maintained at a temperature of 65 °C, and the stirring speed was 150 rpm (magnetic stirrer). The deposition was performed using a pulsed current under optimized conditions (the direct, cathodic pulse current density was −1 mA/cm^2^ (1 s), and the reverse anodic pulse current density was 0.033 mA/cm^2^ (2 s). The whole process contained 900 repeats, which corresponds to a total time of 2700 s.

### 3.4. Synthesis and Application of PEG-b-PCL Copolymer

Sn(Oct)_2_ (tin(II) 2-ethylhexanoate, 0.00725 mM) (Sigma-Aldrich, USA), mPEG 750 (0.0145 mM) (Sigma Aldrich, USA), and toluene (36 mL) (Sigma Aldrich, USA) were mixed at room temperature (RT) for 20 min. Then, ε-caprolactone (1.64 mM) (Sigma-Aldrich, USA) was added to the solution and mixed for 30 min. After mixing, the reaction tube was immersed in an oil bath at 130 °C, and polymerization continued for 48 h with constant stirring. The mixture was precipitated in methanol, redissolved in tetrahydrofuran (Chempur, Poland), and precipitated in methanol again. A total of 0.15 g of copolymer was dissolved in 100 mL ethanol (Avantor Performance Materials, Poland). The PEG-*b*-PCL layer was deposited by drop-coating (4 drops of the volume of 2 µL each) on NiTi/HA, which corresponds to 12 µL/cm^2^ of the PEG-*b*-PCL layer on HA.

### 3.5. Ibuprofen Deposition

A total of 0.15 g of ibuprofen (Cayman Chemical, Ann Arbor, MI, USA) was dissolved in 0.5 mL of ethanol and mixed with 1 mL of PEG-*b*-PCL copolymer. Subsequently, the layer of ibuprofen/PEG-*b*-PCL was drop-coated on HA/PEG-*b*-PCL (10 droplets of volume 2 µL each), which corresponds to 4 mg/cm^2^.

### 3.6. Thickness and Profile Measurements

The profile and thickness of the deposited HA layer were determined using an LSM-6100 laser micrometer with an LSM-500 H scanning head (Mitutoyo, Tokyo, Japan). The measurement was carried out over the entire length of the deposited layer with a lateral resolution of 10 µm and a vertical resolution of 10 nm.

### 3.7. Releasing of Ibuprofen from HA/PEG-b-PCL

The UV-VIS measurements were carried out on the HALO DB 20S UV-VIS Spectrometer (Dynamica, Geneve, Switzerland) in the spectral range 240–290 nm, 100 nm/min.

The calibration curve was prepared according to the Polish Pharmacopoeia [66]. For this purpose, ibuprofen dilutions were prepared in 4% NaOH, and UV-VIS absorbance measurements were performed.

Samples with the HA/PEG-*b*-PCL layer were immersed in 3 mL PBS and incubated at 37 °C, and UV-VIS absorbance was measured after 20 min. Samples were immersed again, and UV-VIS measurements were repeated after 40, 60, 120, 180, 240, and 300 min.

### 3.8. SEM-EDS

The measurements were performed on the Vega LMU Tescan Scanning Electron Microscope (TESCAN, Brno, Czech Republic) using the Oxford Instruments EDS detector. In all cases, the following parameters were used: a beam energy of 20 keV, a probe current of 10 pA, a working distance of 10 mm, a measurement duration of 40 s, and a tilt of 0°. The surface morphology was investigated for surface area of 100∙× 100 µm. The EDS measurements were performed for at least 6 points for each sample.

### 3.9. TOF-SIMS (Time-of-Flight Secondary Ion Mass Spectrometry)

TOF-SIMS spectra were acquired by means of the TOF-SIMS.5 instrument (ION-TOF GmbH, Münster, Germany). The primary ion source of Bi+ was used at 30 keV, primary beam current 1.2 pA, and cyclic time 100 µs. The scanning area of the secondary ions was 100 × 100 µm with 128 × 128 pixels and 1 shot/pixel. All the measurements were performed in a static mode (dose no larger than 1 × 1012 ions/cm^2^) in a negative mode. Data postprocessing was carried out by SurfaceLab 6.7 (ION-TOF GmbH, Münster, Germany).

### 3.10. Raman Spectroscopy

Raman spectra were acquired by means of Raman via microscopy (Renishaw, Sheffield, UK). Measurements were carried out using objective 50x and a 514 nm (Argon, Renishaw, Sheffield, UK) laser source with a power of 30 mW on the sample, an exposure time of 2 s, and 3 acquisitions. Raman spectra were recorded over a range of 190–1390 cm^−1^.

### 3.11. AFM (Atomic Force Microscopy)

AFM measurements were performed on an Agilent 5600LS microscope (Agilent Technologies, Santa Clara, CA, USA) in tapping mode with a silicon tip (radius 7 nm). Measurements were taken for a surface of 20 × 20 and 5 × 5 µm. Roughness calculations were conducted by using SPIP v.5.1.4 (Image Metrology, Lyngby, Denmark).

### 3.12. XPS (X-Ray Photoelectron Spectroscopy)

The XPS measurements were performed on PHI5700/660 Physical Electronics spectrometer (Physical Electronics, Chanhassen, MN, USA) using an Al Kα monochromatic X-ray source with energy 1486.6 eV. All photoelectron spectra were calibrated against the peaks of Au 4f_7/2_ at 83.98 eV, Ag 3d_5/2_ at 368.27 eV, and Cu 2p_3/2_ at 932.67 eV of binding energy. The NiTi wires were measured from a small area with a circle diameter of 400 μm with a pass energy of 23.5 eV. The XPS measurement was carried out for the core lines of O1s, Ni2p, Ti2p, and C1s. Atomic concentration calculations and fitting processes were performed with the use of MULTIPAK (9.9.1) software from Physical Electronics and SIMPEAK software (Physical Electronics, USA).

## 4. Conclusions

The actual studies present the impact of nitinol surface preparation for corrosion properties, the electrodeposition of the hydroxyapatite layer, and subsequent load drug delivery matrix PEG-*b*-PCL for ibuprofen release into PBS solution.

The main results can be summarized as follows:

1. Chemical etching and thermal treatment significantly change the corrosion properties of nitinol. The best anti-corrosion properties reveal nitinol chemically treated and annealed at 470 and 590 °C. It is expressed by the lowest corrosion current and corrosion rates.
2. Morphological studies exhibit the lowest porosity for nitinol thermally treated at 470 °C. Under these conditions, the thinnest TiO_2_ layer among all thermally treated samples in a rutile state is formed.
3. The XPS and TOF-SIMS results reveal the lowest amount of nickel for the sample treated at 470 °C. Due to the potential possibility of releasing nickel ions into the patient’s body and the lowest corrosion current, heat treatment at 470°C seems to be the optimal condition for preparing the NiTi substrate for subsequent deposition of the hydroxyapatite layer and loading the PEG-b-PCL polymer matrix for the release of ibuprofen.
4. The morphology of the electrodeposited hydroxyapatite layer significantly replicates the roughness of the nitinol surface. The lowest porosity (roughness) of nitinol annealed at 470 °C is replicated for the lowest roughness (porosity) hydroxyapatite layer.
5. The PEG-*b*-PCL-deposited layer on the hydroxyapatite layer reveals the highest homogeneity for the layer deposited on nitinol treated at 470 °C. It strongly suggests that a homogenous and less porous hydroxyapatite layer exhibits better absorption of polymeric matrix.
6. Ibuprofen was effectively loaded onto the HA/PEG-*b*-PCL layer in the amount of 2 mg/cm^2^. The ibuprofen release experiment carried out under sink conditions showed that about 70% of ibuprofen is released into PBS within 5 h for thermally treated samples. On the other hand, the amount of released ibuprofen from the HA/PEG-*b*-PCL layer deposited on untreated and etched nitinol is significantly lower (30% for chemically etched and 50% for untreated nitinol, respectively).
7. For the potential application of such a kind of implant, a 5 h release time of ibuprofen would be optimal for reducing pain or inflammation symptoms.
8. Considering corrosion properties, the amount of nickel content on the outermost layer of nitinol surface, and ibuprofen release efficiency from the HA/ PEG-b-PCL bilayer, nitinol surface preparation by chemical etching and subsequent thermal heating at 470 °C under air condition is the most optimal.

## Figures and Tables

**Table 1:** Elemental compositions determined by XPS.

	Atomic %				Mass %	
	Ti	Ni	O	Ti	Ni	O
NiTi	13.67	10.22	76.1	25.7	27.0	47.3
HF	20.86	6.59	72.54	39.1	13.6	47.3
HF_470	29.23	0.44	70.33	54.8	1.1	44.1
HF_570	28.61	1.13	70.25	52.5	3.7	43.8
HF_610	26.93	1.31	71.76	50.8	4.0	45.2

**Table 2:** Corrosion parameters determined for NiTi immersed in PBS solution (pH 7.2).

	Corrosion Potential E_c_ [V]	Corrosion Current Density j_c_ [A/cm^2^]	β_c_ [V]	Corrosion Rate [mm/year]
NiTi	−0.152	4.41 × 10^−8^	0.058	0.0005
HF	−0.302	4.26 × 10^−8^	0.091	0.0005
HF_470	−0.308	1.88 × 10^−8^	0.048	0.0002
HF_590	−0.344	2.08 × 10^−8^	0.059	0.0002
HF_610	−0.339	4.26 × 10^−8^	0.035	0.0005

**Table 3:** Composition of hydroxyapatite layer (oxygen content was omitted) determined by SEM/EDS.

	Average % Mass		Ratio
	P	Ca	Ca:P
NiTi	13.35	21.89	1.64
HF	19.94	33.536	1.68
HF_470	19.67	33.45	1.70
HF_570	20.54	38.04	1.85
HF_610	21.69	37.52	1.73

## Data Availability

The TOF-SIMS, AFM, XPS, and Raman data can be obtained upon reasonable request.

## Supplementary Materials

The following supporting information can be downloaded at: https://www.mdpi.com/article/10.3390/molecules29215200/s1, Figure S1: AFM microimages (20x20 µm^2^) for: (a) untreated NiTi; (b) etched in HF/HNO_3_/H_2_O solution (abbrev. HF); (c) etched in HF/HNO_3_/H_2_O solution and heated at 470°C through 30 min (HF 470); (d) etched and heated at 590°C (HF 590); (e) etched and heated at 610°C (HF 610); Figure S2: AFM microimages (5x5 µm^2^) for: (a) untreated NiTi; (b) etched in HF/HNO_3_/H_2_O solution (abbrev. HF), (c) etched in HF/HNO_3_/H_2_O solution and heated at 470°C through 30 min (HF 470); (d) etched and heated at 590°C (HF 590); (e) etched and heated at 610°C (HF 610); Figure S3: The dependence of OCP (open circuit potential) after immersing in PBS solution for untreated NiTi, etched in HF/HNO_3_/H_2_O solution (abbrev. HF), etched in HF/HNO_3_/H_2_O solution and heated at 470°C through 30 min (HF 470°C), etched and heated at 590°C (HF 590°C), and etched and heated at 610°C (HF 610°C), Figure S4: UV-VIS curves for ibuprofen released from hybrid layer HA/PEG-*b*-PCL deposited on: NiTi (untreated); etched in HF/HNO_3_/H_2_O solution (abbrev. HF), etched in HF/HNO_3_/H_2_O solution and heated at 470°C through 30 min (HF 470); etched and heated at 590°C (HF 590); etched and heated at 610°C (HF 610), Figure S5: Reference curve for Ibuprofen releasing, Figure S6: Cumulative release % of ibuprofen from HA/PEG-*b*-PCL deposited on HF/HA with refresh PBS solution after each measurement.
