# Supplementary material for: Influence of Heat Treatment of Nitinol Wire on the Properties of Nitinol/Hybrid Layer for Ibuprofen Release
Source: Molecules. 2024 Nov 3;29(21):5200. doi: 10.3390/molecules29215200 (PMC11547518; doi:10.3390/molecules29215200)
Supplement: Supplementary file 1 [file molecules-29-05200-s001.zip › molecules-3279446-supplementary.pdf]

Supplementary materials

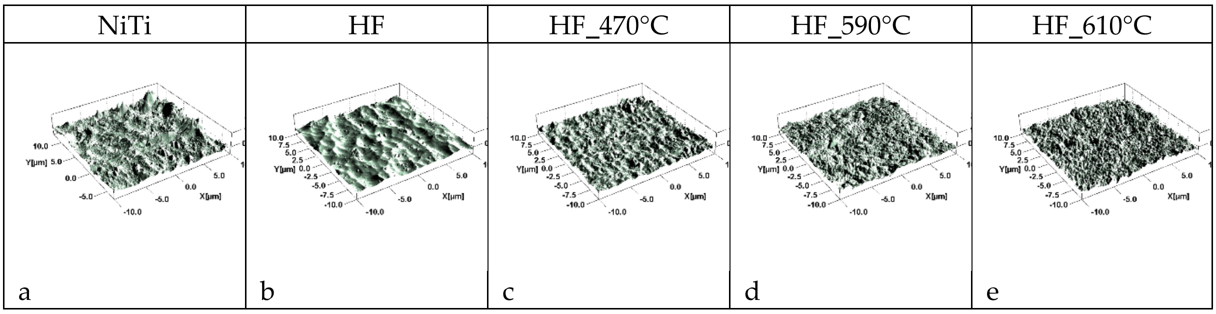

Figure S1. AFM microimages (20x20 μm^2) for: (a) untreated NiTi; (b) etched in $HF/HNO_3/H_2O$ solution (abbrev. HF); (c) etched in $HF/HNO_3/H_2O$ solution and heated at 470°C through 30 min (HF 470); (d) etched and heated at 590°C (HF 590); (e) etched and heated at 610°C (HF 610).

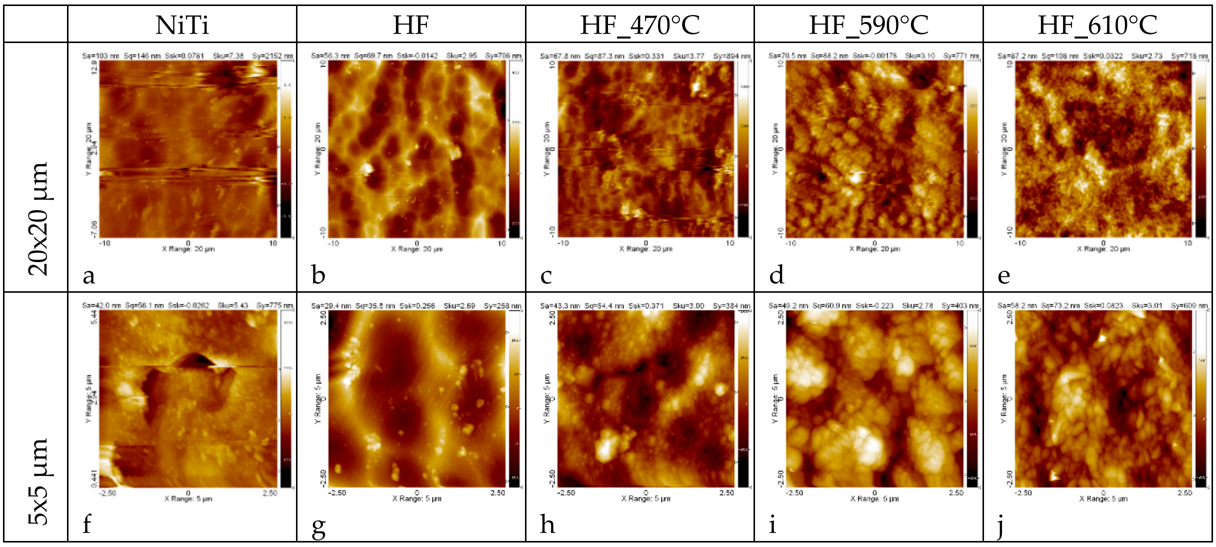

Figure S2. AFM microimages (5x5 μm^2) for: (a) untreated NiTi; (b) etched in $HF/HNO_3/H_2O$ solution (abbrev. HF), (c) etched in $HF/HNO_3/H_2O$ solution and heated at 470°C through 30 min (HF 470); (d) etched and heated at 590°C (HF 590); (e) etched and heated at 610°C (HF 610).

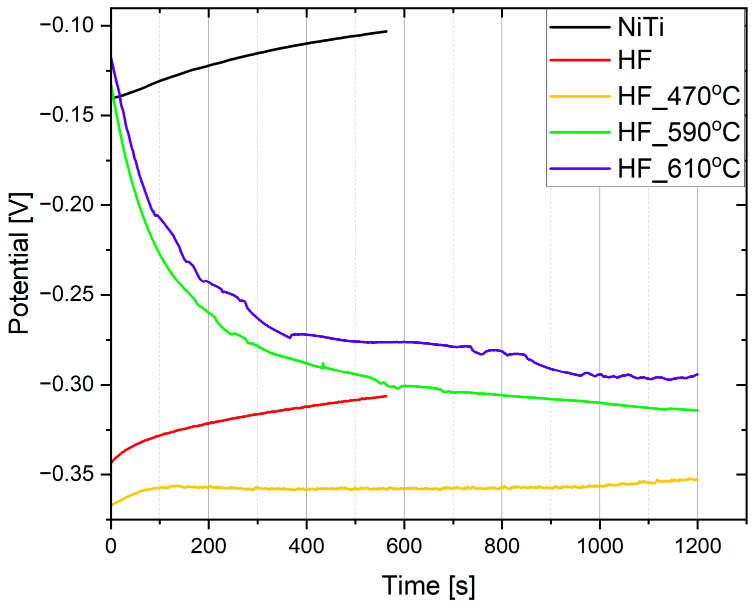

Figure S3. The dependence of OCP (open circuit potential) after immersing in PBS solution for untreated NiTi, etched in $HF/HNO_3/H_2O$ solution (abbrev. HF), etched in $HF/HNO_3/H_2O$ solution and heated at 470°C through 30 min (HF 470°C), etched and heated at 590°C (HF 590°C), and etched and heated at 610°C (HF 610°C).

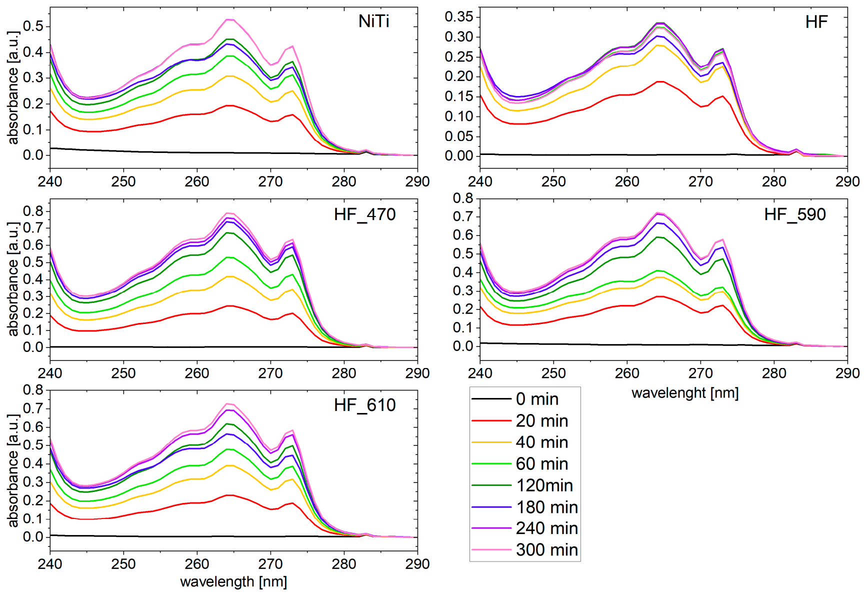

Figure S4. UV-VIS curves for ibuprofen released from hybrid layer HA/PEG-*b*-PCL deposited on: NiTi(untreated); etched in $HF/HNO_3/H_2O$ solution (abbrev. HF), etched in $HF/HNO_3/H_2O$ solution and heated at 470°C through 30 min (HF 470); etched and heated at 590°C (HF 590); etched and heated at 610°C (HF 610).

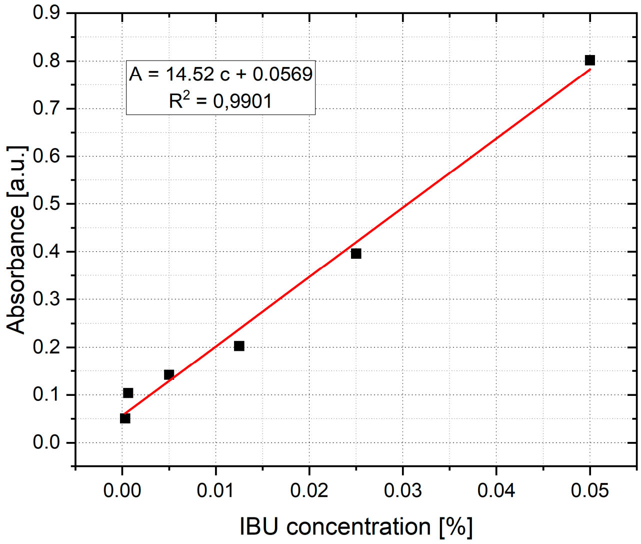

Figure S5 Reference curve for Ibuprofen releasing.

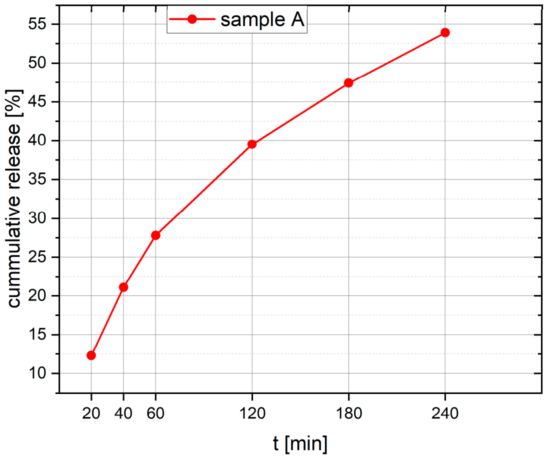

Figure S6. Cumulative release % of ibuprofen from HA/PEG-*b*-PCL deposited on HF/HA with refresh PBS solution after each measurement.
